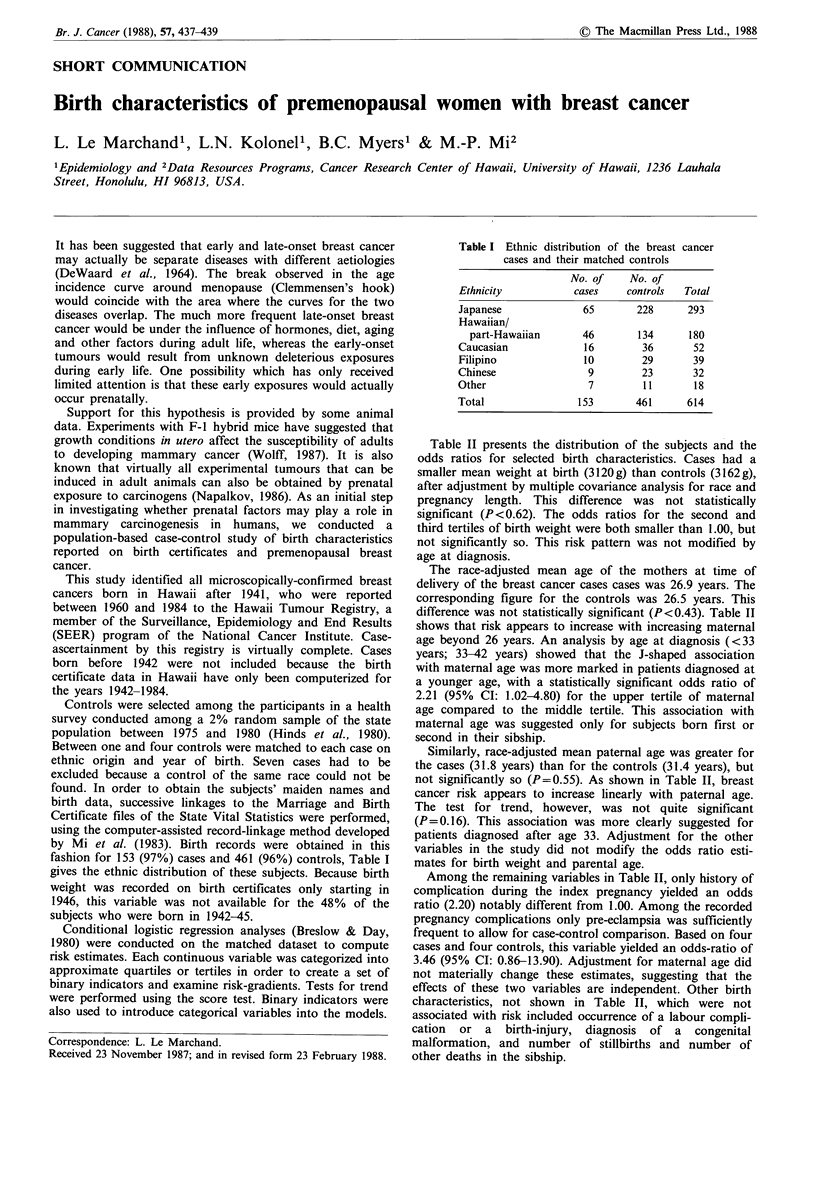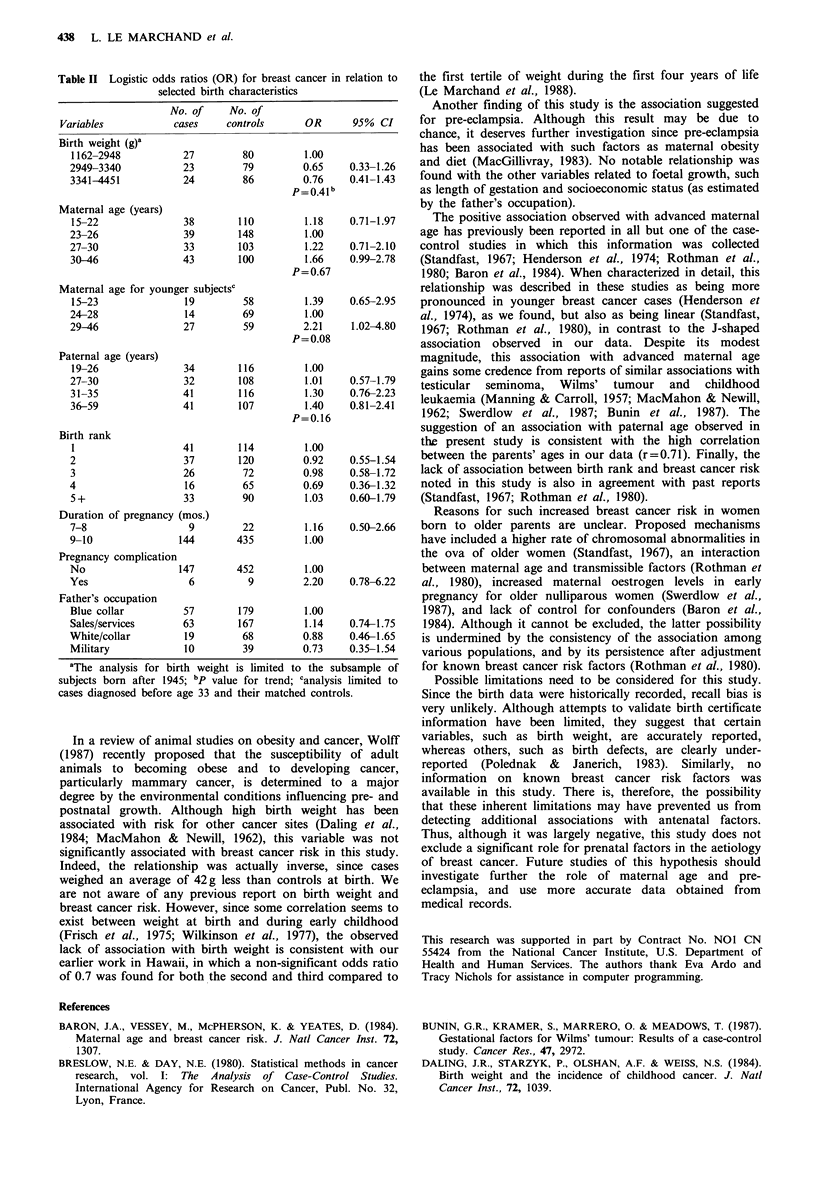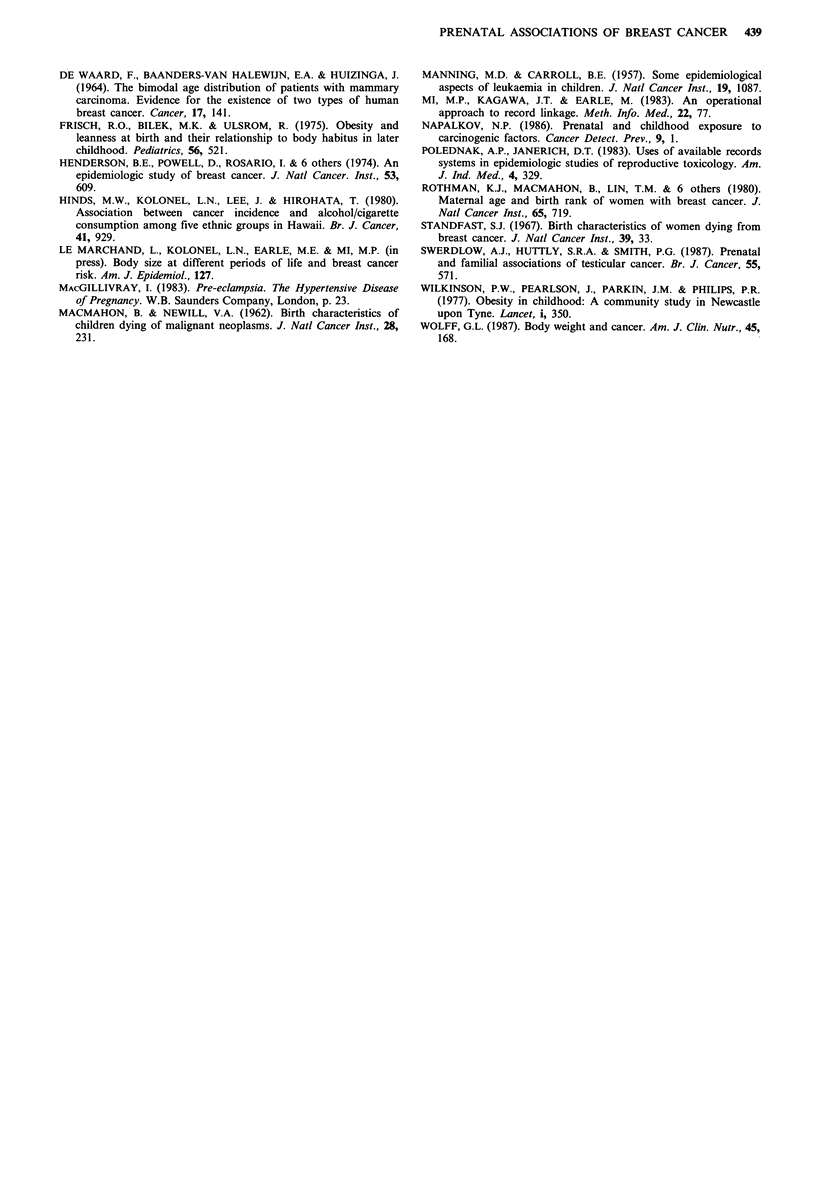# Birth characteristics of premenopausal women with breast cancer.

**DOI:** 10.1038/bjc.1988.99

**Published:** 1988-04

**Authors:** L. Le Marchand, L. N. Kolonel, B. C. Myers, M. P. Mi

**Affiliations:** Epidemiology Program, Cancer Research Center of Hawaii, University of Hawaii, Honolulu 96813.


Br. J. Cancer (1988), 57, 437–439 © The Macmillan Press Ltd., 1988

SHORT COMMUNICATION

### Birth characteristics of premenopausal women with breast cancer

L. Le Marchand[1], L.N. Kolonel[1], B.C. Myers[1] & M.-P. Mi[2]

[1]*Epidemiology and* [2]*Data Resources Programs, Cancer Research Center of Hawaii, University of Hawaii, 1236 Lauhala Street, Honolulu, HI 96813, USA.*

It has been suggested that early and late-onset breast cancer may actually be separate diseases with different aetiologies (DeWaard *et al.*, 1964). The break observed in the age incidence curve around menopause (Clemmensen's hook) would coincide with the area where the curves for the two diseases overlap. The much more frequent late-onset breast cancer would be under the influence of hormones, diet, aging and other factors during adult life, whereas the early-onset tumours would result from unknown deleterious exposures during early life. One possibility which has only received limited attention is that these early exposures would actually occur prenatally.

Support for this hypothesis is provided by some animal data. Experiments with F-1 hybrid mice have suggested that growth conditions *in utero* affect the susceptibility of adults to developing mammary cancer (Wolff, 1987). It is also known that virtually all experimental tumours that can be induced in adult animals can also be obtained by prenatal exposure to carcinogens (Napalkov, 1986). As an initial step in investigating whether prenatal factors may play a role in mammary carcinogenesis in humans, we conducted a population-based case-control study of birth characteristics reported on birth certificates and premenopausal breast cancer.

This study identified all microscopically-confirmed breast cancers born in Hawaii after 1941, who were reported between 1960 and 1984 to the Hawaii Tumour Registry, a member of the Surveillance, Epidemiology and End Results (SEER) program of the National Cancer Institute. Case-ascertainment by this registry is virtually complete. Cases born before 1942 were not included because the birth certificate data in Hawaii have only been computerized for the years 1942–1984.

Controls were selected among the participants in a health survey conducted among a 2% random sample of the state population between 1975 and 1980 (Hinds *et al.*, 1980). Between one and four controls were matched to each case on ethnic origin and year of birth. Seven cases had to be excluded because a control of the same race could not be found. In order to obtain the subjects' maiden names and birth data, successive linkages to the Marriage and Birth Certificate files of the State Vital Statistics were performed, using the computer-assisted record-linkage method developed by Mi *et al.* (1983). Birth records were obtained in this fashion for 153 (97%) cases and 461 (96%) controls, Table I gives the ethnic distribution of these subjects. Because birth weight was recorded on birth certificates only starting in 1946, this variable was not available for the 48% of the subjects who were born in 1942–45.

Conditional logistic regression analyses (Breslow & Day, 1980) were conducted on the matched dataset to compute risk estimates. Each continuous variable was categorized into approximate quartiles or tertiles in order to create a set of binary indicators and examine risk-gradients. Tests for trend were performed using the score test. Binary indicators were also used to introduce categorical variables into the models.

Correspondence: L. Le Marchand.
Received 23 November 1987; and in revised form 23 February 1988.

**Table I** Ethnic distribution of the breast cancer cases and their matched controls

| *Ethnicity* | *No. of cases* | *No. of controls* | *Total* |
|---|---|---|---|
| Japanese | 65 | 228 | 293 |
| Hawaiian/ part-Hawaiian | 46 | 134 | 180 |
| Caucasian | 16 | 36 | 52 |
| Filipino | 10 | 29 | 39 |
| Chinese | 9 | 23 | 32 |
| Other | 7 | 11 | 18 |
| Total | 153 | 461 | 614 |

Table II presents the distribution of the subjects and the odds ratios for selected birth characteristics. Cases had a smaller mean weight at birth (3120 g) than controls (3162 g), after adjustment by multiple covariance analysis for race and pregnancy length. This difference was not statistically significant ($P<0.62$). The odds ratios for the second and third tertiles of birth weight were both smaller than 1.00, but not significantly so. This risk pattern was not modified by age at diagnosis.

The race-adjusted mean age of the mothers at time of delivery of the breast cancer cases cases was 26.9 years. The corresponding figure for the controls was 26.5 years. This difference was not statistically significant ($P<0.43$). Table II shows that risk appears to increase with increasing maternal age beyond 26 years. An analysis by age at diagnosis (<33 years; 33–42 years) showed that the J-shaped association with maternal age was more marked in patients diagnosed at a younger age, with a statistically significant odds ratio of 2.21 (95% CI: 1.02–4.80) for the upper tertile of maternal age compared to the middle tertile. This association with maternal age was suggested only for subjects born first or second in their sibship.

Similarly, race-adjusted mean paternal age was greater for the cases (31.8 years) than for the controls (31.4 years), but not significantly so ($P=0.55$). As shown in Table II, breast cancer risk appears to increase linearly with paternal age. The test for trend, however, was not quite significant ($P=0.16$). This association was more clearly suggested for patients diagnosed after age 33. Adjustment for the other variables in the study did not modify the odds ratio estimates for birth weight and parental age.

Among the remaining variables in Table II, only history of complication during the index pregnancy yielded an odds ratio (2.20) notably different from 1.00. Among the recorded pregnancy complications only pre-eclampsia was sufficiently frequent to allow for case-control comparison. Based on four cases and four controls, this variable yielded an odds-ratio of 3.46 (95% CI: 0.86–13.90). Adjustment for maternal age did not materially change these estimates, suggesting that the effects of these two variables are independent. Other birth characteristics, not shown in Table II, which were not associated with risk included occurrence of a labour complication or a birth-injury, diagnosis of a congenital malformation, and number of stillbirths and number of other deaths in the sibship.

**Table II** Logistic odds ratios (OR) for breast cancer in relation to selected birth characteristics

| *Variables* | *No. of cases* | *No. of controls* | *OR* | *95% CI* |
|---|---|---|---|---|
| Birth weight (g)[a] | | | | |
| 1162–2948 | 27 | 80 | 1.00 | |
| 2949–3340 | 23 | 79 | 0.65 | 0.33–1.26 |
| 3341–4451 | 24 | 86 | 0.76 | 0.41–1.43 |
| | | | *P*=0.41[b] | |
| Maternal age (years) | | | | |
| 15–22 | 38 | 110 | 1.18 | 0.71–1.97 |
| 23–26 | 39 | 148 | 1.00 | |
| 27–30 | 33 | 103 | 1.22 | 0.71–2.10 |
| 30–46 | 43 | 100 | 1.66 | 0.99–2.78 |
| | | | *P*=0.67 | |
| Maternal age for younger subjects[c] | | | | |
| 15–23 | 19 | 58 | 1.39 | 0.65–2.95 |
| 24–28 | 14 | 69 | 1.00 | |
| 29–46 | 27 | 59 | 2.21 | 1.02–4.80 |
| | | | *P*=0.08 | |
| Paternal age (years) | | | | |
| 19–26 | 34 | 116 | 1.00 | |
| 27-30 | 32 | 108 | 1.01 | 0.57–1.79 |
| 31-35 | 41 | 116 | 1.30 | 0.76–2.23 |
| 36–59 | 41 | 107 | 1.40 | 0.81–2.41 |
| | | | *P*=0.16 | |
| Birth rank | | | | |
| 1 | 41 | 114 | 1.00 | |
| 2 | 37 | 120 | 0.92 | 0.55–1.54 |
| 3 | 26 | 72 | 0.98 | 0.58–1.72 |
| 4 | 16 | 65 | 0.69 | 0.36–1.32 |
| 5+ | 33 | 90 | 1.03 | 0.60–1.79 |
| Duration of pregnancy (mos.) | | | | |
| 7–8 | 9 | 22 | 1.16 | 0.50–2.66 |
| 9–10 | 144 | 435 | 1.00 | |
| Pregnancy complication | | | | |
| No | 147 | 452 | 1.00 | |
| Yes | 6 | 9 | 2.20 | 0.78–6.22 |
| Father's occupation | | | | |
| Blue collar | 57 | 179 | 1.00 | |
| Sales/services | 63 | 167 | 1.14 | 0.74–1.75 |
| White/collar | 19 | 68 | 0.88 | 0.46–1.65 |
| Military | 10 | 39 | 0.73 | 0.35–1.54 |

[a]The analysis for birth weight is limited to the subsample of subjects born after 1945; [b]*P* value for trend; [c]analysis limited to cases diagnosed before age 33 and their matched controls.

In a review of animal studies on obesity and cancer, Wolff (1987) recently proposed that the susceptibility of adult animals to becoming obese and to developing cancer, particularly mammary cancer, is determined to a major degree by the environmental conditions influencing pre- and postnatal growth. Although high birth weight has been associated with risk for other cancer sites (Daling *et al.*, 1984; MacMahon & Newill, 1962), this variable was not significantly associated with breast cancer risk in this study. Indeed, the relationship was actually inverse, since cases weighed an average of 42 g less than controls at birth. We are not aware of any previous report on birth weight and breast cancer risk. However, since some correlation seems to exist between weight at birth and during early childhood (Frisch *et al.*, 1975; Wilkinson *et al.*, 1977), the observed lack of association with birth weight is consistent with our earlier work in Hawaii, in which a non-significant odds ratio of 0.7 was found for both the second and third compared to the first tertile of weight during the first four years of life (Le Marchand *et al.*, 1988).

Another finding of this study is the association suggested for pre-eclampsia. Although this result may be due to chance, it deserves further investigation since pre-eclampsia has been associated with such factors as maternal obesity and diet (MacGillivray, 1983). No notable relationship was found with the other variables related to foetal growth, such as length of gestation and socioeconomic status (as estimated by the father's occupation).

The positive association observed with advanced maternal age has previously been reported in all but one of the case-control studies in which this information was collected (Standfast, 1967; Henderson *et al.*, 1974; Rothman *et al.*, 1980; Baron *et al.*, 1984). When characterized in detail, this relationship was described in these studies as being more pronounced in younger breast cancer cases (Henderson *et al.*, 1974), as we found, but also as being linear (Standfast, 1967; Rothman *et al.*, 1980), in contrast to the J-shaped association observed in our data. Despite its modest magnitude, this association with advanced maternal age gains some credence from reports of similar associations with testicular seminoma, Wilms' tumour and childhood leukaemia (Manning & Carroll, 1957; MacMahon & Newill, 1962; Swerdlow *et al.*, 1987; Bunin *et al.*, 1987). The suggestion of an association with paternal age observed in the present study is consistent with the high correlation between the parents' ages in our data (r=0.71). Finally, the lack of association between birth rank and breast cancer risk noted in this study is also in agreement with past reports (Standfast, 1967; Rothman *et al.*, 1980).

Reasons for such increased breast cancer risk in women born to older parents are unclear. Proposed mechanisms have included a higher rate of chromosomal abnormalities in the ova of older women (Standfast, 1967), an interaction between maternal age and transmissible factors (Rothman *et al.*, 1980), increased maternal oestrogen levels in early pregnancy for older nulliparous women (Swerdlow *et al.*, 1987), and lack of control for confounders (Baron *et al.*, 1984). Although it cannot be excluded, the latter possibility is undermined by the consistency of the association among various populations, and by its persistence after adjustment for known breast cancer risk factors (Rothman *et al.*, 1980).

Possible limitations need to be considered for this study. Since the birth data were historically recorded, recall bias is very unlikely. Although attempts to validate birth certificate information have been limited, they suggest that certain variables, such as birth weight, are accurately reported, whereas others, such as birth defects, are clearly under-reported (Polednak & Janerich, 1983). Similarly, no information on known breast cancer risk factors was available in this study. There is, therefore, the possibility that these inherent limitations may have prevented us from detecting additional associations with antenatal factors. Thus, although it was largely negative, this study does not exclude a significant role for prenatal factors in the aetiology of breast cancer. Future studies of this hypothesis should investigate further the role of maternal age and pre-eclampsia, and use more accurate data obtained from medical records.

This research was supported in part by Contract No. NO1 CN 55424 from the National Cancer Institute, U.S. Department of Health and Human Services. The authors thank Eva Ardo and Tracy Nichols for assistance in computer programming.

### References

BARON, J.A., VESSEY, M., McPHERSON, K. & YEATES, D. (1984). Maternal age and breast cancer risk. *J. Natl Cancer Inst.* **72**, 1307.

BRESLOW, N.E. & DAY, N.E. (1980). Statistical methods in cancer research, vol. I: *The Analysis of Case-Control Studies.* International Agency for Research on Cancer, Publ. No. 32, Lyon, France.

BUNIN, G.R., KRAMER, S., MARRERO, O. & MEADOWS, T. (1987). Gestational factors for Wilms' tumour: Results of a case-control study. *Cancer Res.*, **47**, 2972.

DALING, J.R., STARZYK, P., OLSHAN, A.F. & WEISS, N.S. (1984). Birth weight and the incidence of childhood cancer. *J. Natl Cancer Inst.*, **72**, 1039.

DE WAARD, F., BAANDERS-VAN HALEWIJN, E.A. & HUIZINGA, J. (1964). The bimodal age distribution of patients with mammary carcinoma. Evidence for the existence of two types of human breast cancer. *Cancer*, **17**, 141.

FRISCH, R.O., BILEK, M.K. & ULSROM, R. (1975). Obesity and leanness at birth and their relationship to body habitus in later childhood. *Pediatrics*, **56**, 521.

HENDERSON, B.E., POWELL, D., ROSARIO, I. & 6 others (1974). An epidemiologic study of breast cancer. *J. Natl Cancer. Inst.*, **53**, 609.

HINDS, M.W., KOLONEL, L.N., LEE, J. & HIROHATA, T. (1980). Association between cancer incidence and alcohol/cigarette consumption among five ethnic groups in Hawaii. *Br. J. Cancer*, **41**, 929.

LE MARCHAND, L., KOLONEL, L.N., EARLE, M.E. & MI, M.P. (in press). Body size at different periods of life and breast cancer risk. *Am. J. Epidemiol.*, **127**.

MACGILLIVRAY, I. (1983). *Pre-eclampsia. The Hypertensive Disease of Pregnancy*. W.B. Saunders Company, London, p. 23.

MACMAHON, B. & NEWILL, V.A. (1962). Birth characteristics of children dying of malignant neoplasms. *J. Natl Cancer Inst.*, **28**, 231.

MANNING, M.D. & CARROLL, B.E. (1957). Some epidemiological aspects of leukaemia in children. *J. Natl Cancer Inst.*, **19**, 1087.

MI, M.P., KAGAWA, J.T. & EARLE, M. (1983). An operational approach to record linkage. *Meth. Info. Med.*, **22**, 77.

NAPALKOV, N.P. (1986). Prenatal and childhood exposure to carcinogenic factors. *Cancer Detect. Prev.*, **9**, 1.

POLEDNAK, A.P., JANERICH, D.T. (1983). Uses of available records systems in epidemiologic studies of reproductive toxicology. *Am. J. Ind. Med.*, **4**, 329.

ROTHMAN, K.J., MACMAHON, B., LIN, T.M. & 6 others (1980). Maternal age and birth rank of women with breast cancer. *J. Natl Cancer Inst.*, **65**, 719.

STANDFAST, S.J. (1967). Birth characteristics of women dying from breast cancer. *J. Natl Cancer Inst.*, **39**, 33.

SWERDLOW, A.J., HUTTLY, S.R.A. & SMITH, P.G. (1987). Prenatal and familial associations of testicular cancer. *Br. J. Cancer*, **55**, 571.

WILKINSON, P.W., PEARLSON, J., PARKIN, J.M. & PHILIPS, P.R. (1977). Obesity in childhood: A community study in Newcastle upon Tyne. *Lancet*, **i**, 350.

WOLFF, G.L. (1987). Body weight and cancer. *Am. J. Clin. Nutr.*, **45**, 168.